# Ibuprofen-Loaded Chitosan–Lipid Nanoconjugate Hydrogel with Gum Arabic: Green Synthesis, Characterisation, In Vitro Kinetics Mechanistic Release Study and PGE2 Production Test

**DOI:** 10.3390/gels7040254

**Published:** 2021-12-08

**Authors:** Syed Mahmood, Samah Hamed Almurisi, Khater AL-Japairai, Ayah Rebhi Hilles, Walla Alelwani, Azzah M. Bannunah, Farhan Alshammari, Fawaz Alheibshy

**Affiliations:** 1 Department of Pharmaceutical Technology, Faculty of Pharmacy, Universiti Malaya, Kuala Lumpur 50603, Malaysia; 2 Centre for Natural Products Research and Drug Discovery (CENAR), Universiti Malaya, Kuala Lumpur 50603, Malaysia; 3 Department of Pharmaceutical Technology, Kulliyyah of Pharmacy, International Islamic University Malaysia (IIUM), Kuantan 25200, Malaysia; samahhamed8611@gmail.com; 4 Department of Pharmaceutical Engineering, Faculty of Chemical and Process Engineering Technology, Universiti Malaysia Pahang, Gambang 26300, Malaysia; khater.11@hotmail.com; 5 International Institute for Halal Research and Training (INHART), International Islamic University Malaysia, Kuala Lumpur 53100, Malaysia; ayah.hilles90@gmail.com; 6 Department of Biochemistry, Collage of Science, University of Jeddah, Jeddah 21577, Saudi Arabia; welwani@uj.edu.sa; 7 Department of Basic Sciences, Common First Year Deanship, Umm Al-Qura University, Makkah 24230, Saudi Arabia; ambannunah@uqu.edu.sa; 8 Department of Pharmaceutics, College of Pharmacy, University of Hail, Hail 2240, Saudi Arabia; frh.alshammari@uoh.edu.sa (F.A.); fa.alheibshy@uoh.edu.sa (F.A.); 9 Department of Pharmaceutics, College of Pharmacy, Aden University, Aden 6075, Yemen

**Keywords:** ibuprofen, chitosan, green synthesis, transdermal, nanoconjugate, hydrogel

## Abstract

Ibuprofen is a well-known non-steroidal anti-inflammatory (NSAID) medicine that is often used to treat inflammation in general. When given orally, it produces gastrointestinal issues which lead to lower patient compliance. Ibuprofen transdermal administration improves both patient compliance and the efficacy of the drug. Nanoconjugation hydrogels were proposed as a controlled transdermal delivery tool for ibuprofen. Six formulations were prepared using different compositions including chitosan, lipids, gum arabic, and polyvinyl alcohol, through ionic interaction, maturation, and freeze–thaw methods. The formulations were characterised by size, drug conjugation efficiency, differential scanning calorimetry (DSC), and Fourier transform infrared spectroscopy (FTIR). Further analysis of optimised hydrogels was performed, including X-ray diffraction (XRD), rheology, gel fraction and swelling ability, in vitro drug release, and in vitro macrophage prostaglandin E2 (PGE_2_) production testing. The effects of ibuprofen’s electrostatic interaction with a lipid or polymer on the physicochemical and dissolution characterisation of ibuprofen hydrogels were evaluated. The results showed that the S3 (with lipid conjugation) hydrogel provided higher conjugation efficiency and prolonged drug release compared with the S6 hydrogel.

## 1. Introduction

Ibuprofen (IBU) is a crystal powder with low solubility, belonging to class II in the biopharmaceutics classification system (BCS). It exhibits a short half-life (2 h), and rapid clearance following oral administration, leading to a requirement for high and multiple dosing and resulting in missed medication doses and severe side effects [1,2]. In addition, gastric irritation, hepatic first-pass metabolism, and poor patient compliance are all difficulties associated with oral administration [3]. As a result, there is great interest in developing transdermal IBU dosage forms to avoid the adverse effects of oral administration. However, the drug must permeate the uppermost barrier, the stratum corneum, in order to reach appropriate drug levels for treatment effectiveness inside specified skin layers or systemically. Research efforts are currently being focused on the drug conjugate concept, which involves covalently linking drugs to various natural or synthetic molecule carriers such as polymers, polypeptides or proteins, lipids, and carbohydrates for specific applications [4].

The goal of conjugation is to improve the end products’ physicochemical features and therapeutic quality while increasing medication delivery efficiency. In the study conducted by Satheeshababu et al. [5], carvedilol’s drug permeation and bioavailability were significantly improved by bypassing the extensive hepatic first-pass metabolism of the drug through conjugation of the drug with chitosan. The ionised species of IBU (weak organic acid amphiphile) have a propensity for electrostatic interaction that provides a strong affinity for the oppositely charged molecule and hydrogen bonding capacity [6,7]. Furthermore, the hydrogels can be either physically or chemically cross-linked and are rendered insoluble due to the presence of these chemical (covalent or ionic) or physical (freeze/thaw approach) cross-links. As a physical method, the latter has the distinct benefit of cross-linking the polymer solutions [8]. As a result, the medicine is released from the hydrogel with a controlled and sustained release profile, providing patients with long-term relief [9].

The present work focuses on designing conjugates for IBU using Phospholipon 90G (PC90G), and chitosan (CS) with some modification of polyvinyl alcohol (PVA) through the freeze-thaw cycle, as well as using gum arabic (GA) to prepare a hydrogel with desirable properties. The nanoconjugate hydrogel product fabricated using a green approach could assist in enhancing the drug release of IBU through the skin, thus overcoming the limitations of the oral dosage form.

## 2. Results and Discussion

### 2.1. Particle Size Analysis

**Figure 1:**
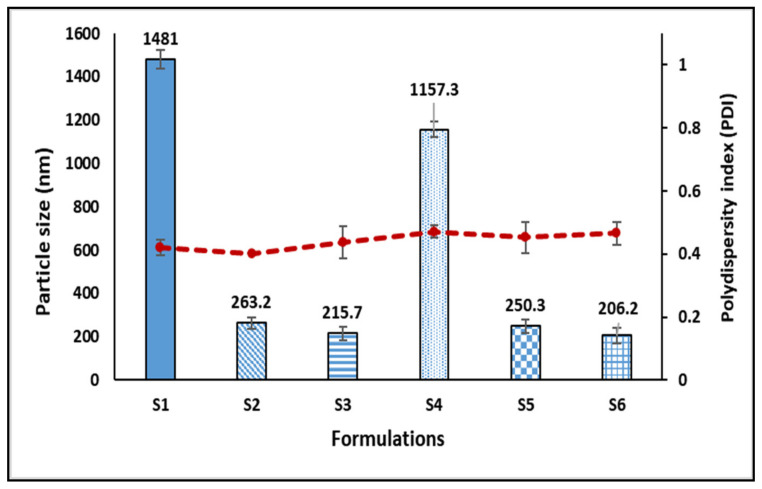
The particle sizes and PDI values for IBU nanoconjugates S1–S6.

All the prepared formulations showed a mean particle size ranging from 206 to 1481 nm (Figure 1). The size of transdermal drug delivery particles has been demonstrated to significantly impact how medications are delivered into the skin. Particles of a diameter of 600 nm or greater cannot carry encapsulated substances into the deeper layers of the skin and are more likely to remain in or on the stratum corneum [10,11,12]. Therefore, an average particle size below 300 nm is ideal for transdermal delivery, to improve the permeation across the stratum corneum [13]. Regarding the formulations prepared, the S1 and S4 formulations cannot be considered to be nanoparticles (as they are above 1000 nm), and this might be attributed to the low ratio of PVA in the formulation. An increased ratio of PVA from 0.5 to 0.75, regardless of lipid, leads to significant reduction in the particle size to less than 300 nm, as seen in S2, S3, S5, and S6. Furthermore, the PDI indicates the uniformity of droplet sizes within the formulation [13]. Although a lower PDI can lead to particle monodispersity, PDI values >0.5 often indicate a larger size distribution [13]. The PDI value of all formulations was found to be lower than 0.5, indicating an acceptable distribution of droplet sizes. The formulations that combined lower particle size and acceptable PDI were S3 and S6.

### 2.2. Conjugation Efficiency Analysis

The conjugation efficiencies of IBU-loaded chitosan–PC90G with gum arabic (S1–S3) and IBU-loaded chitosan with gum arabic (S4–S6) were obtained as recorded in Table 1. The lipid conjugation in S1–S3 plays a significant role in the conjugation efficiency, which was above 90% for these formulations. Conjugation occurs because the bioactives are covalently or non-covalently coupled to a lipidic component in lipid drug conjugates [14]. In addition, the lipid (Phospholipon^®®^ 90G) enriched with lecithin can produce a stiff and stable nanostructure with chitosan through hydrogen bonding and physical entanglement between lecithin and chitosan [15,16,17].

The conjugation efficiencies of S4–S6 were above 80% due to the electrostatic interaction between IBU and chitosan [18]. Ionised IBU species (surface-active molecules) can adsorb onto polymers via hydrophobic and electrostatic bonds (conjugation) with their aromatic ring and hydrophilic carboxylic groups. At the same time, chitosan is cationic and polysaccharides containing a non-polar region and ammonium groups are reported to provide hydrogen bonding capacity and high affinity for oppositely charged molecules [19]. Therefore, the high-density positive charges of chitosan were expected to interact with negatively charged IBU species to produce a drug–polymer complex [19].

Furthermore, PVA includes hydroxyl groups (functional groups) that easily cross-link to produce a hydrogel by repeated freezing and thawing cycles [20]. In addition, polysaccharides in chitosan and gum arabic can readily bind to PVA due to the high number of polar carboxyl and hydroxyl groups, giving rise to hydrogen bonding [21]. Moreover, it was reported that PVA hydrogel could be physically cross-linked with IBU via repeated freeze–thaw cycling and gamma irradiation [22]. Comparing the formulations, it was found that the formulations containing lipid had a higher conjugation efficiency.

### 2.3. Fourier Transform Infrared Spectroscopy (FTIR)

**Figure 2:**
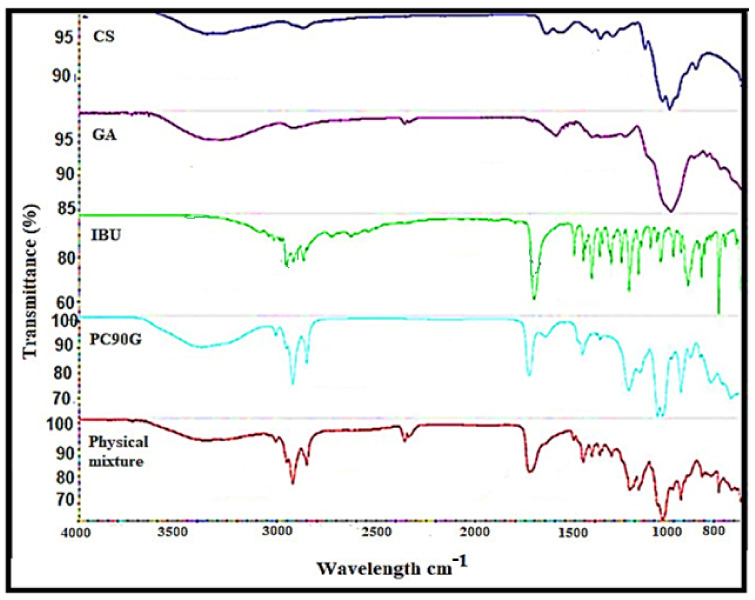
FTIR spectra of chitosan (CS), gum arabic (GA), pure ibuprofen (IBU), PC90G (lipid) and physical mixture.

Figure 2 shows the FTIR spectra of pure IBU, chitosan powder, gum arabic powder, Phospholipon 90G lipid, and a physical mixture of the drug with excipients. In the spectrum of IBU, there was an O–H stretching peak at 2954.28 cm^−1^, which was the major characteristic peak of the carboxylic acid group of IBU [18]. In addition, there was an asymmetrical peak at 1708.5 cm^−1^ (strong, sharp) from the carbonyl group (C=O), as well as bands at 1507.05 cm^−1^ (strong, sharp) and 1230.45 cm^−1^ due to aromatic ring vibration (C=C) and (C–O) stretching, for IBU [18].

In the case of the lipid PC90G, peaks appeared for the stretching band of the ester carbonyl group (C=O stretching) at 1735.34 cm^−1^, together with an ester C–O stretching band at 1232.06 cm^−1^, and C–H stretching bands at 2923.07 cm^−1^ and 2853.06 cm^−1^ [23]. For the chitosan spectrum, there were significant peaks at 3355.17 cm^−1^ (N–H stretching and O–H stretching) corresponding to its amine group and at 1644.94 cm^−1^, 1374.04 cm^−1^, and 1317.07 cm^−1^ (N–H bending and C=O stretching) corresponding to its amide I group, a vibration of the protonated amine group and amide III, respectively [24]. The absorption bands at 1150.72 cm^−1^ are characteristic of vibration of the CO group (C–O stretching and C–N stretching) in its saccharide structure [18].

The gum arabic showed a broad absorption band at 3311.30 cm^−1^, attributed to stretching due to –OH groups, and three peaks at 1601.06 cm^−1^, 1540.57 cm^−1^, and 1417.70 cm^−1^ corresponding to the asymmetrical and symmetrical stretching of –COO- groups [25]. Furthermore, in the physical mixture, the characteristic carboxylic group of IBU was shifted to the upper wavenumber, from 1708 cm^−1^ to 1733 cm^−1^, which was attributed to the participation of this functional group in the interaction between IBU and Phospholipon 90G [26]. In general, the FTIR analysis of the physical mixture showed a slight shifting of frequency for IBU peaks, which might be related to IBU’s electrostatic interaction with the lipid or polymer or both via hydrogen bonding between IBU’s carboxylic group and chitosan’s protonated amino group.

### 2.4. Differential Scanning Calorimetry (DSC) Analysis

**Figure 3:**
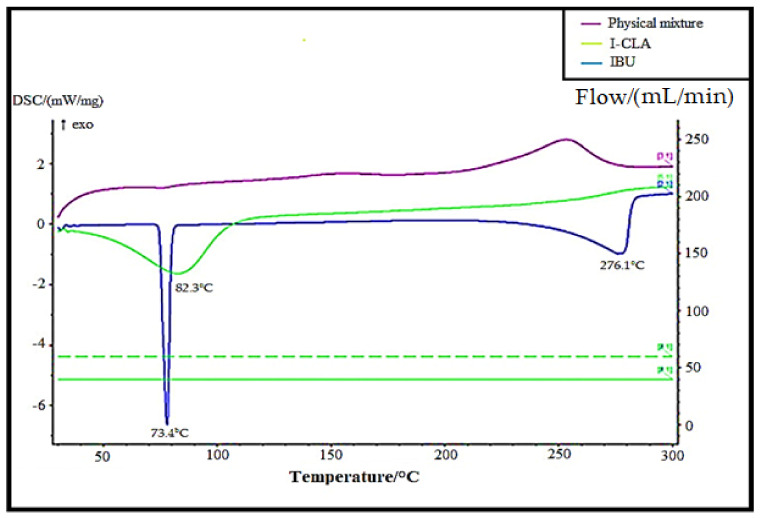
DSC thermograms of pure IBU, physical mixture, and IBU-loaded chitosan–PC90G with gum arabic (I-CLA).

The DSC thermograms of pure IBU were compared with the physical mixture of IBU with excipients and IBU-loaded chitosan–PC90G with gum arabic (I-CLA), as shown in Figure 3. The thermogram of IBU was typical of a crystalline anhydrous substance, with a sharp endothermic peak at 73.4 °C indicating its melting point [27] and a second peak occurring at around 276.1 °C, which is due to the endothermic evaporation of ibuprofen [28]. On the other hand, the I-CLA thermogram shows a broad endothermic peak at 82.3 °C that was attributed to a transition from the solid crystal to the liquid crystal state for the corresponding compounds. The sharp endothermic peak of IBU disappeared in the physical mixture of ibuprofen with excipients, indicating that the IBU converted to an amorphous state. These DSC results are in agreement with a study performed earlier, where amorphous ibuprofen was prepared in Upsalite (a mesoporous magnesium carbonate) [29].

### 2.5. HPLC Standard Curve

**Figure 4:**
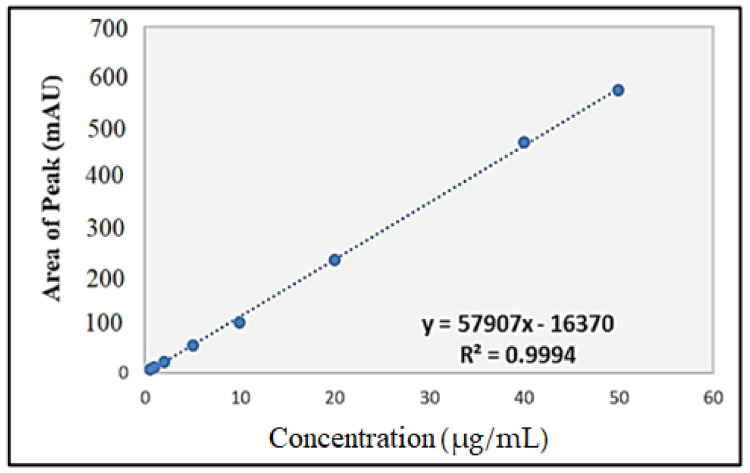
Standard curve of ibuprofen for HPLC analysis.

The standard curve of IBU (Figure 4) was plotted based on the areas of the peaks against the concentration of IBU solutions. The relationship was found to be linear within the concentration range of 0.5–50 µg/mL for IBU, with a regression equation of y = 57,907x − 16,370 that showed an excellent correlation coefficient of 0.9994, where y and x represent the area under the curve and the concentration of IBU in µg/mL, respectively. The limit of detection (LOD) and the limit of quantification (LOQ) were 0.02 µg/mL and 0.05 µg/mL, respectively.

### 2.6. X-ray Diffraction Analysis

**Figure 5:**
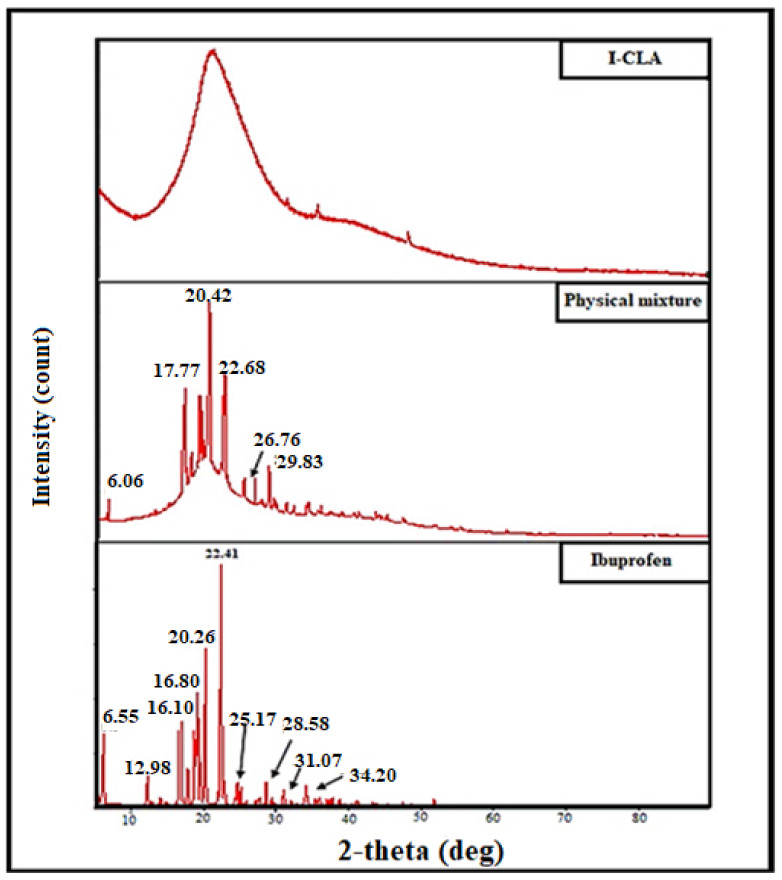
XRD patterns of pure ibuprofen powder, physical mixture, and IBU-loaded chitosan–PC90G with gum arabic (I-CLA).

The XRD analysis examined the amorphous or crystalline form of pure IBU, a physical mixture of the drug and excipients, and IBU-loaded chitosan–PC90G with gum arabic (I-CLA), as shown in Figure 5. Pure IBU exhibits a crystalline structure with sharp diffraction peaks. These peaks were reduced in the physical mixture of IBU with excipients. On the other hand, the peaks in the XRD pattern of I-CLA disappeared and were replaced by diminished and broader peaks. The broader peak could be attributed to the formation of a high-energy amorphous state of IBU. However, there are some IBU-specific diffraction peaks, indicating the existence of some IBU crystals in the I-CLA formulation. In general, the disappearance of crystalline peaks, shifts, decreases in peak intensity, or the emergence of new peaks in XRD patterns are typically due to amorphisation and/or complexation [30]. Both the DSC and XRD results suggested that the drug was molecularly dispersed or in an amorphous form, which is favourable for enhancing the dissolution rate for poorly soluble drugs such as IBU.

### 2.7. Rheology

**Figure 6:**
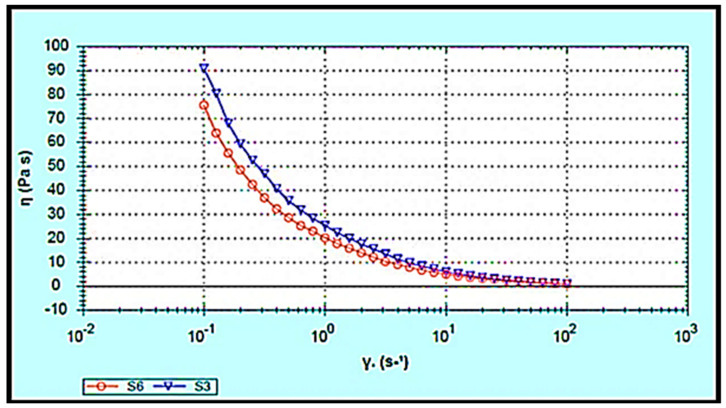
Flow curve of ibuprofen-loaded chitosan-PC90G with gum arabic (S3) and ibuprofen-loaded chitosan with gum arabic (S6). Viscosity vs. shear rate.

The rheological behaviour of the hydrogels was investigated since it affects the materials’ flow characteristics during the mixing process, packaging into containers, storage, and application to the skin. Furthermore, drug release from semisolid formulations is influenced by the rheological properties [31,32]. The apparent viscosities (Pa.s) of S3 and S6 were measured as a function of shear rate (1/s). The S3 and S6 hydrogels showed non-Newtonian pseudoplastic flow or shear-thinning behaviour. The viscosity decreased with respect to the shear rate in both S3 and S6 hydrogels, fitting a power-law model with a high regression value (r = 0.99), as illustrated in Figure 6. This behaviour of S3 and S6 hydrogels contributes to their spreadability when applied to the skin surface. Ideally, the hydrogel should have sufficient viscosity to be applied and to spread smoothly across the skin without slipping off under minor gravitational force [33].

**Figure 7:**
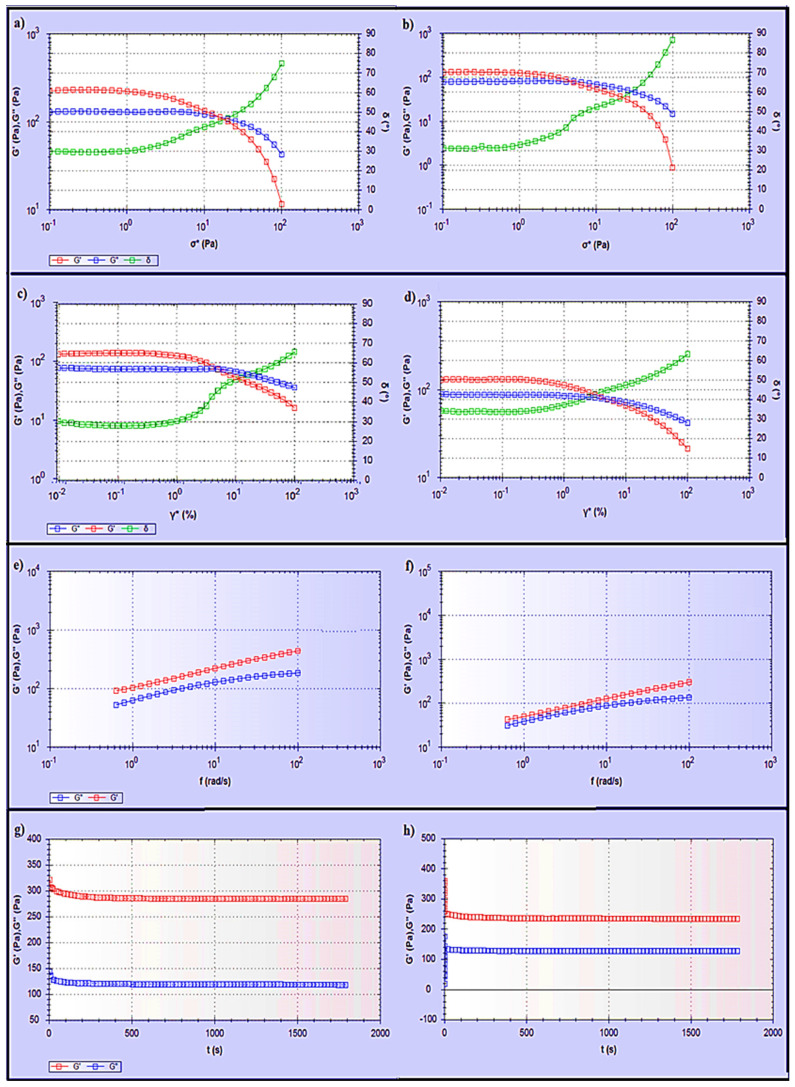
Rheological study of (**a**) stress sweep test for S3, (**b**) stress sweep test for S6, (**c**) dynamic strain sweep test for S3, (**d**) dynamic strain sweep test for S6, (**e**) dynamic frequency sweep test for S3, (**f**) dynamic frequency sweep test for S6, (**g**) dynamic time sweep test for S3, (**h**) dynamic time sweep test for S6.

Additionally, products intended for transdermal use are subjected to shearing forces (oscillatory test) that represent the hydrogel rubbing over the skin and the skin’s flexing processes [33]. Hydrogels are a type of viscoelastic system that displays both solid- and liquid-like behaviour, commonly analysed by the assessment of the elastic modulus (G′), which defines the solid-like character of the material, and the viscous modulus (G″), which describes the liquid-like character. The rheology measurement should be carried out without destroying the hydrogel structure, to quantify the contribution of each modulus. To accomplish this goal, an amplitude sweep test (stress sweep and strain sweep) must be performed to define the linear viscoelastic range (LVR), where G′ and G″ are independent of stress or strain. As shown in Figure 7a–d, the LVR region observed in both hydrogels (the region in which a sample is capable of maintaining its structure when force is applied) provides information on the hydrogel structure/firmness [34]. Moreover, the G′ values were higher than the G″ values, and the phase angle (δ) values were between 0 and 45°. This indicates that both S3 and S6 hydrogels exhibit viscoelastic behaviour. Comparing the S3 and S6 hydrogels showed that S3 exhibited a slightly higher elastic G′ modulus, indicating greater rigidity and stiffness than S6. In the second phase, when the shear stress/shear strain was increased above some critical level, values of both G′ and G″ suddenly changed and the value of the phase angle δ sharply increased and exceeded 45°. Hence, the hydrogels converted to liquid-like behaviour as G″ became higher than G′.

Moreover, a dynamic frequency sweep (DFS) test was carried out to determine the behaviour of the hydrogel at varying frequencies. As frequency is the inverse of time, the samples have time to relax at low frequencies and become more elastic as the frequency increases. Simply, a lower gel strength is associated with G’ being more dependent on frequency [35]. The curves from the dynamic frequency sweep test shown in Figure 7e,f, indicate that the S3 and S6 hydrogels had reasonable gel strength with only a slight dependence of G′ on the applied frequency, and that G′ was larger than G″ over the frequency range. Furthermore, the dynamic time sweep (DTS) test provided information about the behaviour of the material with time. In Figure 7g,h, both S3 and S6 hydrogels show stable behaviour, whereas the elastic modulus (G′) and the viscous modulus (G′′) are unchanged with time.

### 2.8. Gel Fraction and Swelling Ability of the Optimised Hydrogels

In hydrogels, the gel fraction is a key component in determining the degree of crosslinking [36]. As the gel fraction increased, the strength of the gel increased but the flexibility decreased. The gel fractions of the S3 and S6 hydrogels were 81.52 ± 2.1 and 78.5 ± 5.3%. The gel fraction depends on the number of freezing cycles, the PVA content in the blend, and the chitosan content [37]. A high chitosan content leads to a reduced cross-linking reaction between polymers in the hydrogel, decreasing the gel fraction [38]. On the other hand, the gel content increases as the number of freezing cycles and the PVA content in the blend increase. A high quantity of PVA and an increase in the number of freeze-thaw cycles result in a high percentage gel fraction.

The swelling ratio of S3 was 380.4 ± 20.06%, while that of S6 was 409.7± 10.19%. The swelling ratio is influenced by the polymer chain and cross-linking densities; as the polymer chain and cross-linking densities increased, the gel structure became denser, and the swelling ratio decreased [39]. In addition, the swelling percentage declined with a high number of freeze–thaw cycles, which could be attributed to hydrogen bond formation between the amino group in chitosan and the hydroxyl group in PVA macromolecules [37]. The rate of drug release is greatly affected by the swelling of hydrogels; as the swelling of the hydrogel increases, so does the rate of drug release. As a result, the swelling features of hydrogels were examined in order to understand the IBU release mechanism better.

### 2.9. Drug Release of Optimised IBU Hydrogels

**Figure 8:**
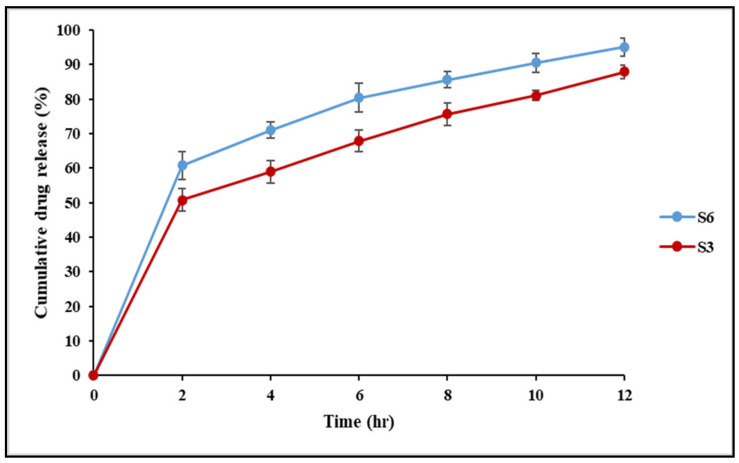
The drug release profile for IBU-loaded chitosan–PC90G with gum arabic (S3) and IBU-loaded chitosan with gum arabic (S6).

Release studies with Franz-type diffusion cells were used to evaluate the ability of the suggested systems to function as carriers for the transdermal drug delivery of IBU. Both S3 and S6 nanoconjugate hydrogels showed an initial burst of release during the first 2 h, followed by a slow and sustained release for 10 h, as illustrated in Figure 8. A rapid initial release of the drug commonly occurs in controlled release systems and is most likely due to drug molecules that are weakly adsorbed on the surface of the hydrogel [40]. The sustained release of the drug could be attributed to the formation of strong interactions in the network structure through the cross-linking process.

Direct drug molecule incorporation into the hydrogel matrix has been reported to result in a very strong initial burst release profile, potentially exceeding 70% of the total drug loading, resulting in a lesser amount of drug being available for extended release [41]. Thus, moderate-to-high-affinity interactions efficiently slow down the drug release process, which is especially useful when a more prolonged release is desired. Controlled drug release over a long period is frequently advantageous for reducing the application frequency and the doses delivered, which enhances patient compliance [42]. IBU is extensively metabolised in the liver following oral administration, and the medicine must be given often because of its short biological half-life. As a result, the transdermal route looks interesting for maintaining IBU blood levels over an extended period of time.

There are various chemical and physical interactions to choose from, ranging from covalent conjugation to secondary interactions such as electrostatics and hydrophobic associations, to achieve these high-affinity interactions. Comparing S3 and S6 nanoconjugate hydrogels, the highest release was obtained in the S6 hydrogel, while S3 gave lower cumulative drug release. The lower release could be attributed to retardation owing to the conjugation of IBU to the phospholipid P90G. The results here agreed with the findings of previous researchers [43], who employed P90G as a retarding agent. Furthermore, as the density of cross-links increases, the swelling of networks diminishes, lowering the release rate.

Chitosan (CS) plays an important role as a rheological modifier in IBU release and its penetration into the receiver part of the Franz cells. In addition, the bioadhesive characteristics of chitosan improve drug retention at the site of application and prolong the release rate of the incorporated drug [44]. A similar result was found in the study conducted by Abioye et al. [18] aimed at developing a chitosan–ibuprofen–gellan nanogel, where the chitosan provides controlled release of IBU in the nanogel. Gum arabic (GA) is another natural polysaccharide obtained from the Acacia tree; it is a class of negatively charged, hydrophilic, non-toxic biopolymers utilised as an additive to improve and diversify the characteristics of chitosan via electrostatic attraction between CS and GA [45]. Chitosan/gum arabic Pickering emulsions were successfully used as biodegradable surfactant-free vehicles for the skin application of trans-resveratrol. The skin permeation results for resveratrol from the developed emulsions showed that resveratrol was retained at higher levels in the viable epidermis and dermis, which is advantageous for giving the required cosmetic effect without the need for using chemical penetration enhancers [46]. Transdermal drug delivery based on polymer–drug conjugates, surface functional groups on polymer carriers, or drug molecules can affect the permeation efficiency. The mammalian skin contains a net negative charge, which attracts positively charged molecules while repelling negatively charged molecules [47]. The IBU nanoconjugate hydrogels prepared in this study (S3 or S6) were prepared by low-energy, ionic interaction, maturation, and modification of the PVA-CS (freeze–thaw) method, which is a safe, environmentally friendly, and green process.

To further understand the drug release mechanism, the data from the drug diffusion study were fitted into a few mathematical models, including zero-order kinetics, first-order kinetics, Higuchi, Korsmeyer–Peppas and Hixson–Crowell models (Table 2). The IBU nanoconjugate hydrogels (S3 and S6) best fitted into the Korsmeyer–Peppas model with r^2^ values of 0.9929 and 0.9950, respectively. Further, the kinetic exponents ‘n’ for S3 and S6 were less than 0.43, indicating that the release followed Fickian diffusion. Thus, the drug was released by a diffusion mechanism from the matrix in a controlled manner. Moreover, the hydrogels followed the first-order rate model more closely than the zero-order rate model, suggesting sustained release behaviour for the hydrogels. Additionally, the times required to release different amounts of IBU (T25%, T50%, T75%, T80%, and T90%) from the nanoconjugate hydrogels (S3 and S6) were obtained from the most suitable kinetic model (the Korsmeyer–Peppas model), as presented in Table 3. The time required to release IBU in S3 was longer than in S6, which suggests that the lipid conjugation provides a more extended release of IBU.

### 2.10. Effect of the Optimised IBU Hydrogels in PGE2 Production

LPS is the main component of the Gram-negative bacteria’s outer membrane, stimulating diverse inflammatory reactions and inducing the release of a related pro-inflammatory mediator such as prostaglandin E2 (PGE2) and inflammatory cytokines [48]. PGE2 is a widely known inflammatory mediator derived from arachidonic acid through the action of cyclooxygenases (COX) [49]. As a result, inhibiting COX-2 expression from limiting PGE2 production could be a primary therapeutic approach in developing anti-inflammatory drugs like IBU that block PGE2 production to relieve inflammation [50].

**Figure 9:**
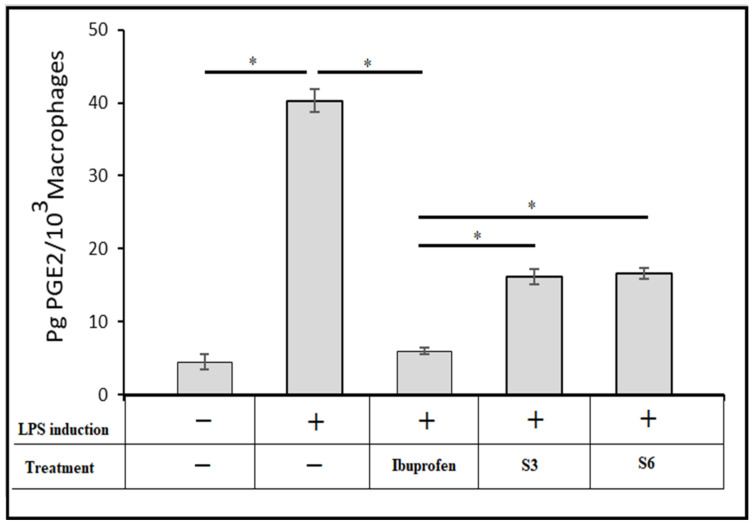
The effect of optimised IBU nanoconjugate hydrogels S3 and S6 on the production of prostaglandin E2 in RAW264.7 cells induced by LPS, where + indicates LPS induction was used to treat the cells,—indicates LPS was not used to treat the cells, and * indicates a *p*-value <0.05 for one-way ANOVA with Tukey’s post hoc test.

The pharmaceutical effects of the ibuprofen-optimised hydrogel formulations S3 and S6 were monitored utilising LPS to stimulate macrophages, as shown in Figure 9. PGE2 levels in the control group (untreated) increased noticeably after 6 h of treatment with 1 mg/mL of LPS. On the other hand, adding pure IBU in equivalent quantities to those existing in the optimised formulations S3 and S6 neutralised the LPS effect in PGE2 production. Furthermore, the IBU-loaded chitosan-PC90G with gum arabic (I-CLA) (S3) and IBU-loaded chitosan with gum arabic produced a decline in PGE2 synthesis after LPS induction. This showed that the inhibitory effect of the chemically modified ibuprofens S3 and S6 on PGE2 production was maintained, although to a lesser extent.

## 3. Conclusions

A simple and rapid technique was used to formulate a hydrogel containing IBU loaded in a chitosan–lipid nanoconjugate for the application of delivering IBU transdermally. The role of lipid or polymer conjugation represents a new trend for the transdermal delivery of active compounds. The hydrogel was prepared using a novel combination of gum arabic with in situ ionic gelation. The sizes of the optimised nanoconjugates S3 and S6 were reported as between 206 and 250 nm, and the conjugation efficiency was more than 91%. In vitro release studies on a cellulose membrane for S3, and S6 nanoconjugate hydrogels showed an initial burst release during the first 2 h. The IBU nanoconjugate hydrogels (S3 and S6) best fitted the Korsmeyer–Peppas model with r^2^ values of 0.9929 and 0.9950, respectively. The nanoconjugate hydrogel I-CLA was able to reduce the crystallinity of IBU and provided extended and controlled release of IBU that facilitated overcoming the issues associated with the oral dosage form. The polymer chain and cross-linking densities influenced the swelling ratio of the formulated hydrogels. The optimised hydrogel showed a viscous behaviour (G″  >  G′), and the rheological behaviour was typical of a “weak gel”. The effect of IBU reduced the PGE2 synthesis after LPS induction. This study showed that such combinations of lipid and polymer show promising results and could be used in the future for specialised drug delivery platforms.

## 4. Materials and Methods

### 4.1. Materials

A hydrogel IBU drug was purchased from Acros Organics (Thermo Fisher Scientific, New Jersey, USA). Chitosan and gum arabic were purchased from R&M Chemicals. Phospholipon 90G (PC-90G) lipid was obtained from Lipoid GmbH, Nattermannallee, Switzerland. Phosphate-buffered saline (PBS) was purchased from Invitrogen. Fetal bovine serum (FBS), and Dulbecco’s Modified Eagle Medium (DMEM) containing 4.5 g/L of glucose and 4 mM L-glutamine were purchased from Invitrogen (Carlsbad, CA, USA). The Human Prostaglandin E2 (PGE2) ELISA kit was purchased from Biorbyt Ltd., Cambridge, U.K. All other chemicals that were used were of analytical grade.

### 4.2. Methods

#### 4.2.1. Formulation of IBU-Loaded Chitosan-PC90G with Gum Arabic (I-CLA) Hydrogel

The protocol of Huang and co-workers (2019) was followed with minor modifications to prepare the hydrogel using the green synthesis approach. The ibuprofen-loaded chitosan-PC90G with gum arabic (I-CPA) hydrogel was formulated using ionic interaction and freeze-thawing of PVA-CS [51].

1. Preparation of IBU-Loaded Chitosan–PC90G Nanoconjugate

**Figure 10:**
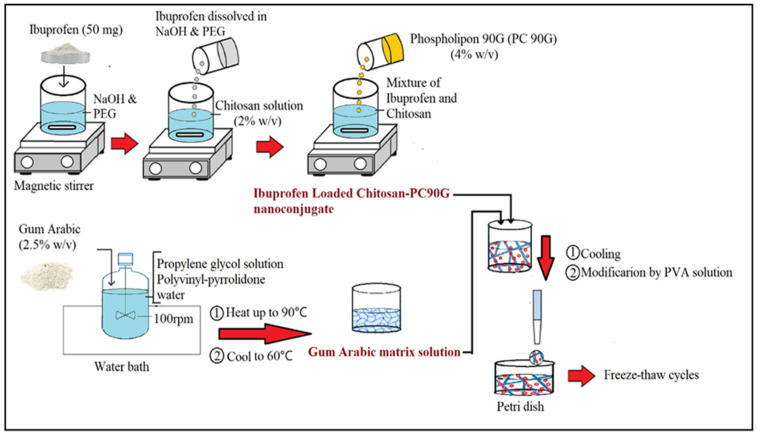
Schematic diagram of the fabrication procedure for IBU nanoconjugate hydrogel.

The IBU-loaded nanoconjugate was prepared in several steps as illustrated in Figure 10. Briefly, 50 mg of IBU was dissolved in 6 mL of sodium hydroxide (NaOH) and 2 mL of polyethene glycol (PEG 400) in the first step. Then, this was made up to 25 mL with distilled water. To prepare a drug–polymer suspension, chitosan (400 mg) was dissolved in 9 mL of 2% acetic acid and made up to 20 mL with distilled water to give a 2% w/v concentration of the chitosan. The IBU solution prepared in the first step was added in drops to the chitosan solution to formulate a drug–polymer suspension. Then, about 4% w/v of PC90G was prepared as the drug conjugation enhancer by dissolving 200 mg of PC90G in 5 mL of methanol, then adding to the mixture of IBU and chitosan. Removal of the methanol solvent was then performed on the IBU-loaded chitosan–lipid conjugates by keeping overnight under a fume hood to remove the residual solvents.

2. Fabrication of Hydrogel with Gum Arabic (Green Approach)

Secondly, the nanoconjugate with a gel matrix of gum arabic was prepared by dispersing about 1.25 gm of gum arabic into 10 mL of 10% propylene glycol solution and 6 mL of 2% polyvinyl-pyrrolidone, under continuous stirring (100 rpm) in a jacketed vessel in a water bath (Hotplate, Thermo Scientific, Cimerax, Germany) for 1 h. The solution of gum arabic was made up with 50 mL of distilled water to obtain a 2.5% w/v concentration of GA, and then heated up to 90 °C to allow gum maturation and to achieve a homogenous consistency, after which it was allowed to cool to 60 °C. Next, the IBU nanoconjugate was incorporated into the gum arabic matrix solution. Finally, after cooling the nanoconjugate in the gel matrix solution, the modification was made by adding a 10% w/v PVA solution at different ratios. The final IBU-loaded nanoconjugate hydrogels were then poured into a Petri dish and underwent freezing at (−80 °C) and thawing (at 25 °C) for a minimum of 6 cycles [52].

There were six formulations from S1 to S6; their differences were in the conjugations with lipid (PC90G) and the ratio of PVA in the formulation, as illustrated in Table 4. The weight of IBU was kept constant for every formulation at 50 mg. In addition, primary characterisations were performed on each formulation to observe the differences and changes in particle size, together with the polydispersity index (PDI) and drug conjugation efficiency.

#### 4.2.2. Characterisation of I-CLA-Mediated Hydrogel

1. Particle Size and Polydispersity Index

A particle size analyser (Malvern, Zetasizer S20, Cambridge, UK) was used to quantify the particle sizes of the formulations. To measure the size and the PDI, 0.1 mL of prepared particles was diluted with 2.0 mL of distilled water (ultrapure), loaded into a disposable cuvette, and scanned for particles. The measurement was done at a 173° scattering angle. The backscattering detection at 173° excludes the excess scattered light and allows detection of the low-intensity scattered light signals originating from tiny particles [53]. The experiment was performed with a temperature setting of 25 ± 0.5 °C. Samples were ultrasonicated to minimise particle interactions and agglomeration before preparation for size measurement. Data were taken for triplicate readings (*n* = 3).

2. Drug Conjugation Efficiency

The conjugation efficiencies of the formulations were determined by calculating the difference between the IBU amounts added and the non-conjugated IBU amounts, using a dialysis bag. The dialysis bag with two ends tied was loaded with 2 mL of formulations and placed into a dissolution apparatus containing 450 mL of distilled water. The distilled water with the tied dialysis bags containing the sample was stirred at 100 rpm at 25 °C [18]. After 3 h, 5 mL of the sample was taken, diluted with a suitable solvent, filtered, and analysed using an HPLC calibration curve for quantifying the drug content. The detected amount of drug was considered to be free or unconjugated drug, and the conjugation efficiency was calculated using the formula as shown in Equation (1).

(1) $$\mathrm{Conjugate}\;\mathrm{efficiency}=\frac{M_{i}-M_{n}}{M_{i}}\times 100\%$$

where Mi is the initial IBU amount added and Mn is the unconjugated IBU amount detected in the dialysis bag after 3 h. The measurements are averages of triplicate readings.

3. Fourier Transform Infrared Spectroscopy

The structural changes due to interactions between IBU, chitosan (CS), PC90G, gum arabic (GA), and a physical mixture including the drug and excipients, were investigated using a PerkinElmer ATR-FTIR spectrometer (L1600401 Spectrum Two DTGS, Llantrisant, UK). In brief, about 10 mg of drug, excipients, and physical mixture were placed on the diamond floor plate of the machine, and sufficient stress was applied to ensure they were firmly clamped. The spectrum scan was performed from 400 to 4000 cm^−1^ wavelengths with an average of 16 scans for each sample.

4. Differential Scanning Calorimetry Analysis

The changes in enthalpy and melting temperature of pure IBU powder, the physical mixture including the drug and excipients, and IBU-loaded chitosan–PC90G with gum arabic (I-CLA) were investigated using a differential scanning calorimeter (DSC 8000, PerkinElmer Inc., Llantrisant, UK). Briefly, samples were placed in aluminium pans that were subsequently crimp-sealed, with a blank aluminium pan serving as a reference. To establish an inert environment, the device was fed a steady stream of nitrogen gas at a rate of 20 mL/min. The samples were heated in the DSC chamber at a heating rate of 10 °C per minute over a temperature range of 30 °C to 300 °C.

5. HPLC Analysis

The drug content in the samples was quantified using an HPLC system (G1311B, Agilent Technologies, Santa Clara, CA, USA), and an end-capped C reverse-phase analytical column was used. The mobile phase comprised potassium phosphate buffer with a pH of 7.0, adjusted with 20 mM sodium hydroxide and HPLC grade acetonitrile (63:37 v/v). IBU was monitored at 233 nm with a flow rate of 1ml/min, a retention time of 6 min and an injection volume of 10 μL.

6. X-ray Diffraction Analysis

X-ray diffraction was utilised to determine the crystallinity behaviour of pure IBU, the physical mixture in powder form, and IBU-loaded chitosan–PC90G with gum arabic (I-CLA) in freeze-dried form. The patterns of crystallinity of the samples were obtained using an X’Pert3 powder X-ray diffractometer (Bruker D8 Advance, New Jersey, USA). The measurement parameters were Cu-K radiation with a 2-theta angle ranging from 5° to 60°, a voltage of 40 kV, and a scanning rate of 10 s per step.

7. Rheological Studies

Rheological measurements were made using a RS6000 rheometer (HAAKE, Karlsruhe, Germany). The conditions included a measuring temperature of 37 °C and a cone rotor (cone angle: 1°, cone diameter: 35 mm) with a gap of 1 mm. The flow behaviour was measured using the viscosity curve, and the graphs were then presented as viscosity (Pa.s) vs. the shear rate function (γ˙) and were fitted to a power-law model [54]. The viscoelastic behaviour of the gels was determined by an oscillatory amplitude sweep test which was carried out at a fixed frequency of 0.623 rad/s with shear stress ranging from 1 Pa to 100 Pa, in the linear viscoelastic region (LVR) of the samples. In addition, a dynamic strain sweep (DSS) was performed at a constant angular frequency of 0.623 rad s^−1^ while altering the applied strain from 0.1 to 100%. The dynamic frequency sweep (DFS) test was conducted at 1% strain within the LVR over the frequency range of 0.628–100 rad/s. Moreover, the dynamic time sweep (DTS) test was performed at a constant shear frequency (0.628 Hz) and constant shear strain (1%) for 30 min, to evaluate the changes in the elastic stored modulus G′ and the viscous loss modulus G′′ with time.

8. Gel Fraction and Swelling Ability of the Optimised Hydrogels

The hydrogels S3 and S6 were freeze-dried and weighed (Wo) before being soaked in distilled water for at least 24 h. The hydrogels were re-weighed after drying to eliminate the soluble components (We). The following equation was used to compute the percentage gel fraction:

(2) $$\mathrm{Gel}\;\mathrm{fraction}\;\left(\%\right)\;=\;\frac{\mathrm{We}}{\mathrm{Wo}}\times 100$$

For the swelling ability test, dried hydrogels (We) were immersed in PBS for 24 h at 37 °C. The hydrogels were then withdrawn from the PBS, and any excess PBS was blotted away with filter paper. The weights of the swollen hydrogels (Ws) were then determined, and the ability of the produced hydrogels to swell was calculated using the following equation:

(3) $$\mathrm{Swelling}\;\mathrm{ratio}\;\left(\%\right)\;=\;\frac{\mathrm{Ws}-\mathrm{We}}{\mathrm{We}}\times 100$$

9. In Vitro Drug Release Study

An in vitro drug release experiment was performed using the Franz diffusion cell method. The Franz diffusion cell includes donor and receptor compartments and a receiver separated by a cellulose membrane. The receptor compartment was filled with 40% (*v*/*v*) ethanol in PBS (pH 7.4) in a ratio (40:60 *v*/*v*) that ensured sink conditions. Its temperature was maintained at 32 ± 0.5 °C to maintain skin surface temperature and mimic in vivo conditions, using a re-circulating water bath. The solution in the receptor chambers was stirred continuously at 100 rpm [55,56]. A quantity of 50 mg of the S3 and S6 formulations tested was placed in the donor compartment and spread evenly on the cellulose membrane. Samples of 400 μL were withdrawn at predetermined time intervals for 12 h and replaced with the same volume of fresh PBS (pH 7.4). The samples were then diluted with HPLC mobile phase, filtered using a 0.45 µm filter and analysed using an HPLC instrument. The concentration of IBU was calculated using the regression equation y = 57907x − 16370 from the standard calibration curve of IBU, where y represents the area under the curve and x represents the concentration of IBU in µg/mL. Following this, the cumulative drug release (%) was calculated based on the following equation:

(4) $$\mathrm{Cumulative}\;\mathrm{drug}\;\mathrm{release}\;\mathrm{of}\;\mathrm{IBU}\;\left(\%\right)\;=\;\frac{\left[\mathrm{IBU}\right]t}{\left[\mathrm{IBU}\right]\mathrm{total}}\times 100$$

where [IBU]t is the amount of IBU released at time t and [IBU]total is the total IBU amount present in the formulation.

The mechanism of drug release was analysed using the release kinetics models, including zero-order, first-order, Hixon–Crowell and Korsmeyer–Peppas models [4]. Furthermore, the equation of the best-fitting model was used to determine T25, T50, T75, T80, and T90% values, which are the times required to dissolve 25, 50, 75, 80, and 90%, respectively, of the drug in the pharmaceutical dosage form.

10. In Vitro Macrophage Prostaglandin E2 (PGE2) Production Test

The reported protocol of Lin et al. (2016) was followed [57]. The RAW264.7 macrophage cell line was utilised to assess the inhibitory effect of the IBU formulations (S3 and S6) on PGE2 production. Briefly, 1 × 10^5^ RAW264.7 macrophage cells were seeded into a six-well plate containing DMEM with 10% of FBS. After 2 h of culture, the medium was switched to the RAW264.7 culture medium (3 mL) (control group), RAW264.7 culture medium containing ibuprofen (50 µM) (IBU group), S3 (IBU-loaded chitosan–PC90G hydrogel with gum arabic), or S6 (IBU-loaded chitosan hydrogel with gum arabic). Twenty-four hours after medium replacement, lipopolysaccharide LPS (3 mL, 1 mg/mL) was introduced, and the medium was harvested 6 h after LPS induction [58]. The level of PGE2 was measured using a Human (Prostaglandin E2) PGE2 ELISA kit (Biorbyt Ltd., Cambridge, UK). Each measurement was carried out three times.

#### 4.2.3. Statistical Analysis

The statistical analysis was performed in triplicate, and the results were reported as a mean and standard deviation (SD). Tukey tests were used to assess the data, followed by ANOVAs. SPSS for Windows was used to conduct the analyses (Chicago, IL, USA). A *p*-value of less than 0.05 was considered statistically significant.

## Figures and Tables

**Table 1:** Conjugation Efficiency of IBU hydrogel with and without lipid.

Formulation Code	Conjugation Efficiency (%)
S1	91.15 ± 0.37
S2	91.93 ± 0.97
S3	91.95 ± 0.92
S4	84.85 ± 0.78
S5	87.05 ± 0.29
S6	88.15 ± 0.41

**Table 2:** Fitted mathematical models for formulated IBU nanoconjugate hydrogels S3 and S6.

Models	Zero Order	First Order	Korsmeyer-Peppas Model		Hixson-Crowell Model
Formulation	r^2^	r^2^	r^2^	n	r^2^
S3	0.5609	0.9257	0.9977	0.320	0.7926
S6	0.4076	0.9545	0.9997	0.253	0.8408

**Table 3:** Time (hours) necessary to release 25, 50, 75, 80, and 90% of IBU loaded in nanoconjugate hydrogels S3 and S6.

Kinetic Parameters	S3	S6
T25%	0.249	0.061
T50%	2.179	0.948
T75%	7.740	4.719
T80%	9.470	6.093
T90%	13.686	9.712

**Table 4:** Optimisation of the IBU nanoconjugate hydrogels. The variation of components determines the changes in particle size, together with the polydispersity index (PDI) and drug conjugation efficiency.

Formula Code	IBU (mg)	CS (w/v)	PC90G	GA (w/v)	PVA (w/v)	PVA: Nanoconjugate Hydrogel Vol.
			(w/v)			Ratio
S1	50	2%	4%	2.50%	10%	0.25:1
S2	50	2%	4%	2.50%	10%	0.5:1
S3	50	2%	4%	2.50%	10%	0.75:1
S4	50	2%	-	2.50%	10%	0.25:1
S5	50	2%	-	2.50%	10%	0.5:1
S6	50	2%	-	2.50%	10%	0.75:1

## Data Availability

None.
